# An Innovative Ultrasonic Apparatus and Technology for Diagnosis of Freeze-Drying Process

**DOI:** 10.3390/s19092181

**Published:** 2019-05-11

**Authors:** Chin-Chi Cheng, Yen-Hsiang Tseng, Shih-Chang Huang

**Affiliations:** 1 Department of Energy and Refrigerating Air-Conditioning Engineering, National Taipei University of Technology, Taipei 10608, Taiwan; albertschuang@gmail.com; 2 Tai Yiaeh Enterprise Co., Ltd., New Taipei City 23942, Taiwan; yhtntut@gmail.com

**Keywords:** freeze-drying process diagnosis, ultrasonic transducer (UT), freezing/drying point, drying period

## Abstract

The freeze-drying process removes water from a product through freezing, sublimation and desorption procedures. However, the extreme conditions of the freeze-drying environment, such as the limited space, vacuum and freezing temperatures of as much as −50 °C, may block the ability to use certain diagnostic sensors. In this paper, an ultrasonic transducer (UT) is integrated onto the bottom of a specially designed frozen bottle for the purpose of observing the freeze-drying process of water at varying amounts. The temperatures and visual observations made with a camera are then compared with the corresponding ultrasonic signatures. Among all of the diagnostic tools and technologies available, only ultrasonic and visual records are able to analyze the entire progression of the freeze-drying process of water. Compared with typical experiment settings, the indication of drying point for water by the amplitude variations of ultrasonic L^3^ echo could reduce the process period and energy consumption. This study demonstrates how an innovative frozen bottle, an integrated ultrasonic sensor and diagnostic methods used to measure and optimize the freeze-drying process of water can save energy.

## 1. Introduction

Dehydration extends food’s usage period longer than that of fresh food by preserving it in a stable and safe condition [1,2]. The conventional methods of drying include solar drying, air drying, spray drying [3], microwave drying [4], infrared drying, fluidized bed drying [5], spouted bed drying, vacuum drying and freeze-drying [6]. Drying methods can be separated into natural and artificial categories. Artificial methods are more advantageous than the natural methods [7] because they can remove large amounts of moisture efficiently by being able to control the different parameters involved such as the temperature, drying air flux and time of drying and so forth. [8]. For long-term storage of food, drug and biopharmaceutical products, most manufacturers utilize the freeze drying process, due to its advantages including better stability, easy handling and storage, as well as better overall product quality [9,10].

The freeze-drying process is comprised of the freezing, primary drying and then secondary drying stages [11] to remove the water from a product. The water contained in food is cooled down and becomes ice during the freezing stage. This stage governs the sublimation and desorption rates and the quality of the lyophilized product [12]. Then, in the primary drying stage, the air in the vacuum chamber is exhausted and the chamber pressure is reduced below the vapor pressure of ice. Meanwhile, the shelf temperature increases gradually to sublimate the ice. The residual water inside the food will be desorbed thoroughly in the secondary drying stage [13]. 

In the freezing stage, the accuracy of the freezing point data is related to the water activity, frozen water [14], freezing and the thawing of frozen food. The freezing point is also important in order to estimate the freezing time, the end point of freezing and the fraction of unfrozen water in food. Then, in the primary drying stage, the chamber pressure and food temperature [15] decides the sublimation rate of the ice, primary drying period, dried pore structure and product quality. A lower product temperature and the corresponding lower vapor pressure of the ice can result in longer primary drying times and higher manufacturing costs [16]. However, if the product temperature increases above the “critical formulation temperature,” this may lead to losing the pore structure, to shrinkage or to fully collapsing. In the secondary drying stage, the product temperature controls the rate of desorption and the obtainable moisture level. Further reduction of the chamber pressure is not necessary in this stage [17]. Secondary drying times are usually designed to achieve a reduction of moisture content within the dried product to less than 1% [18]. Due to the importance of these parameters and its wider applications to the freeze drying process, the estimated freezing/drying points and the ability to model these properties is crucial in food processing (freezing and drying) and food stability during storage [10,19]. 

**Figure 1:**
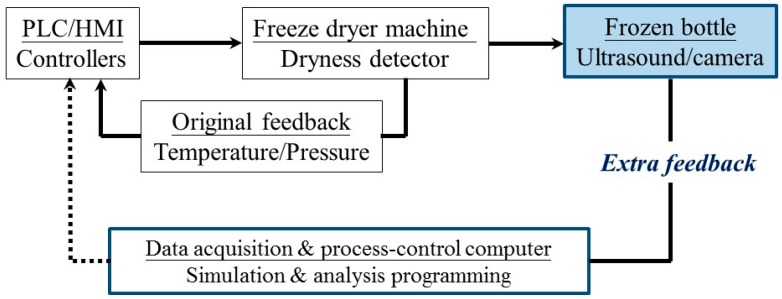
Controlling structure of the freeze-drying process by using the innovative frozen bottle method.

Being able to perform real-time process diagnosis will be beneficial to understanding the complex interplay between the different elements during the freeze-drying process to better enhance quality control procedures. However, the extreme conditions of the freeze-drying environment, such as the limited space, vacuum and freezing temperatures as low as −50 °C, may block the ability to use certain diagnostic sensors [6,20]. The typically used diagnostic tools for real-time measurement of the freeze-drying process are temperature and pressure sensors [20,21]. In order to provide temperature information, a temperature sensor is put in direct contact with a frozen sample [21]. However, inconveniences arise because it is often not easy to remove the sensor after the freeze-drying process. Additionally, using this diagnostic method on drug and biopharmaceutical products may cause undesired contamination due to a sensor being in direct contact with the sample. The chamber pressure is measured by the pressure sensor or vacuum gauge. The measured chamber pressure value cannot directly indicate the physical variation of frozen product real-time during the freeze-drying process [20,22]. In this paper, an idea for diagnosing the physical property variations of a product in the frozen bottle to provide additional information for a machine system is presented, as shown in Figure 1. 

In order to circumvent the limitations of the mentioned sensors and provide real-time process diagnosis, the ultrasonic technique is one of the most widely known non-destructive and non-intrusive methods [23]. The fundamental properties of ultrasonic signals include reflection and transmission coefficients, velocity, attenuation and scatter signals given off from the materials. These signatures have specific relationships with properties of materials [24], process variations [25] and sample quality [26]. Ultrasonic echoes can also detect the temperature [27,28] and pressure [29] of a material. Passot et al. utilized ultrasonic technology to decrease the sublimation time during the primary drying stage by controlling the nucleation temperature [30]. The authors presented an innovatively designed frozen bottle with an ultrasonic transducer and ultrasonic technology to be utilized for the diagnosis of the freezing point during the frozen process in our previous paper [22]. In this study, the ultrasonic technique will be applied for real-time diagnosis of the freeze-drying process. An ultrasonic transducer (UT) is integrated into the bottom of a frozen bottle. During the freeze-drying process at various amounts of water, the process is analyzed by using ultrasonic technology to evaluate the freezing/drying points and the drying period to optimize the process and save energy.

## 2. Design of Frozen Bottle with Ultrasonic Transducer

**Figure 2:**
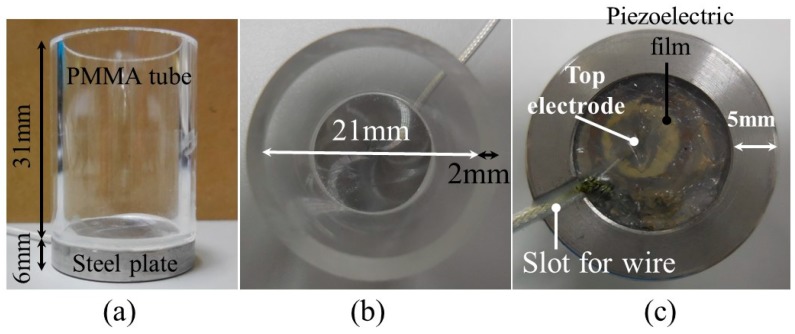
Photographs of the designed apparatus from (**a**) the side, (**b**) the top and (**c**) the bottom view.

In order to solve the aforementioned limitations regarding the transmission of signals in a vacuum, as well as the limitations of being unable to view the process using the steel freezing bottle apparatus or the opaque samples, an innovative freezing bottle apparatus was designed as shown in Figure 2. The innovatively designed container used for freezing is comprised of two parts: a steel plate as the bottom holder and a transparent polymethylmethacrylate (PMMA) tube as the side wall of the container. Figure 2a shows the side view of the frozen bottle. A steel plate with a diameter of 25 mm and a height of 6mm was designed for better heat transmission with the heating/cooling shelf. A transparent PMMA tube measuring 31 mm high, with a diameter of 21 mm and a thickness of 2 mm was designed for better visibility. The steel plate and PMMA tube were then glued together. Figure 2b shows the top view of the apparatus. Figure 2c shows the bottom view of the apparatus. The UT is comprised of Pb(Zr_x_Ti_1−x_)O_3_ (PZT) material and integrated into the cavity of steel plate using the sol-gel spray technique, as described in previous publications [22,24,31]. The operational temperature range of the UT is between −100 and 400 °C.

**Figure 3:**
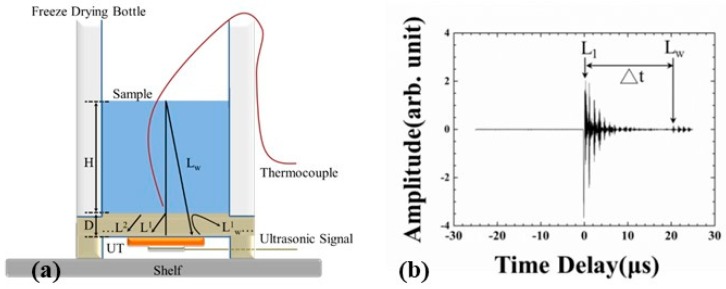
(**a**) Schematic drawing of cross-section of the designed apparatus with an ultrasonic transducer (UT) and thermocouple and (**b**) shows the typical ultrasonic echoes acquired by the UT.

To understand the transmission paths of the ultrasonic signals within the apparatus, a schematic view of the apparatus placed upright is displayed in Figure 3. A temperature sensor (Type T thermocouple) was installed in the middle of the apparatus for measuring the water/ice temperature. The temperature can be recorded and then compared with the ultrasonic signals received during the freeze-drying process. As shown in Figure 3a, an ultrasonic transducer (UT) was integrated onto the bottom of the steel plate. When electric pulses are applied to the piezoelectric film, the ultrasonic signals are transmitted into the steel plate. L^n^ (n = 1, 2,…) denotes the nth round trip of longitudinal ultrasonic echoes reflected from the interface of the steel plate/water or ice and L_w_ is the echo reflected from the water/air interface. The L^3^ and L_w_ echoes are used to monitor the freeze-drying process and water/ice state. The height of the water level and the thickness of the bottom of the apparatus are denoted as H and D, respectively. 

When the water was fed into the apparatus, the typical ultrasonic signals acquired by the UT are presented in Figure 3b. It was observed that the L^1^ echo, which reflected from the steel plate/water or ice interface, appeared as 0.81μs and remained so during the entire process. The operating frequency of the ultrasonic transducer was 9.13 MHz and the −6 dB bandwidth was 8.0 MHz. The signal to noise ratio (SNR) for the ultrasonic L^1^ echo was 38.5 dB. The L_w_ echo, which propagated in the water and reflected from the water/air interface, was observed at 20.39 μs. Following the L^1^ and L_w_ echoes, there were several echoes reflected from the steel plate/water or ice interface. The desired ultrasonic echoes were detected by the acquiring windows of the acquiring system. The time delay difference between the L^1^ and L_w_ echoes is denoted as Δt.

## 3. Experiments

**Figure 4:**
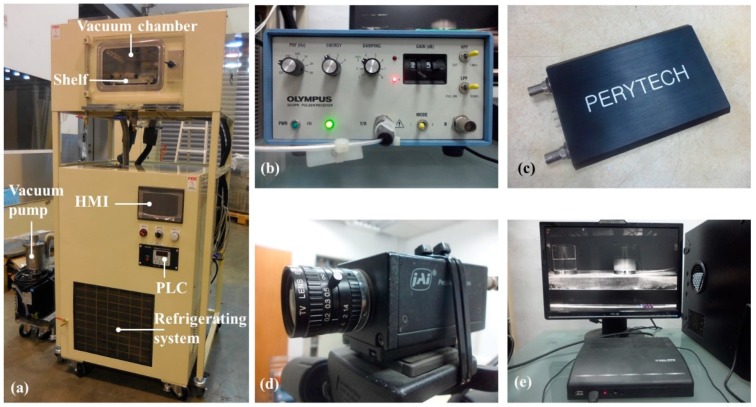
Photographs of (**a**) the split-type freeze-drying machine with vacuum chamber, controller unit, refrigeration unit and vacuum pump, (**b**) the ultrasonic pulse/receive unit, (**c**) a PC-based digital oscilloscope, (**d**) Charge-coupled device (CCD) camera, (**e**) digital video recorder (DVR) and PC.

In order to confirm the hypothesis that the apparatus was able to perform improved diagnostics of the freeze-drying process in a vacuum, a 4 L air-cooled split-type shelf freeze-drying machine, equipped with vacuum chamber, shelf, control units, refrigeration system and vacuum pump, was used to carry out all experiments, as shown in Figure 4a. The vacuum chamber was used for freezing and drying the samples under temperature conditions ranging from +50 to −40 °C and pressures from 760 to 0.05 Torr. The apparatus was set on the shelf of vacuum chamber for the freeze-drying process. The control unit was composed of a programmable logic controller (PLC) and a human-machine interface (HMI) for controlling the freeze-drying process. The measured temperature data from the thermocouple were recorded by the PLC control unit every second during the freeze-drying process. 

The ultrasonic signal is triggered and received by the pulse/receive unit (5072PR, Olympus, Japanese), as shown in Figure 4b. It is equipped with a broadband negative spike pulser and receiver, which can be operated in reflection or transmission mode. All the experiments in this study were carried out using the ultrasonic pulse/receiver unit’s pulse-echo mode. The ultrasonic signals were acquired every second during the entire cycle. The ultrasonic signals received during the freeze-drying process were monitored and recorded with the digital storage USB oscilloscope (DSO-U2400, Perytech, Taipei, Taiwan), as shown in Figure 4c. 

To compare the temperatures and ultrasonic signals, a charge-coupled device (CCD) camera, digital video recorder (DVR) and PC were used to capture dynamic images of the apparatus during the freeze-drying process, as shown in Figure 4d–e. The CCD camera (CV-M10BX, JAI, Japan) is a progressive scan camera with a standard interlaced video output at a resolution compatible with VGA or SVGA formats. The camera was equipped with a 35 mm lens for better resolution. The DVR (08KD, Kingnet, New Taipei City, Taiwan) is an 8-channel recorder that uses the H.264 image compression format. 

In this study, different amounts of water at levels of 5, 10 and 15 mm were put into the apparatus for the duration of the freeze-drying process. The settings of a typical freeze-drying procedure are outlined in Table 1. The shelf temperature was set at −30 °C to freeze the water and the freezing period was 120 min. During the freezing period, the water in the apparatus/container was cooled down and it began to freeze. After enough cooling and freezing, the chamber was exhausted and the chamber pressure was reduced from 760 to 0.17 Torr within several seconds. Then the primary and secondary drying processes were started and the ice sublimated into gas continuously. The shelf temperature was incrementally increased by 10 degree intervals from −30 to 10 °C at heating and pausing periods of 60 and 120 min, respectively. The pausing period of 0 °C is 300 min to sublimate ice further. During the sublimation of the ice, the chamber pressure was reduced further to 0.036 Torr.

## 4. Results and Discussions

For exploring the physical phenomena that occur during the freeze-drying process and searching for the indicators of freezing or drying completeness, the measured temperature and pressure in the vacuum chamber, ultrasonic echoes and velocity of the water/ice and visual observation during the freeze-drying process are analyzed in the following sections. 

### 4.1. Temperature and Pressure Variations in the Vacuum Chamber during the Freeze-Drying Process 

**Figure 5:**
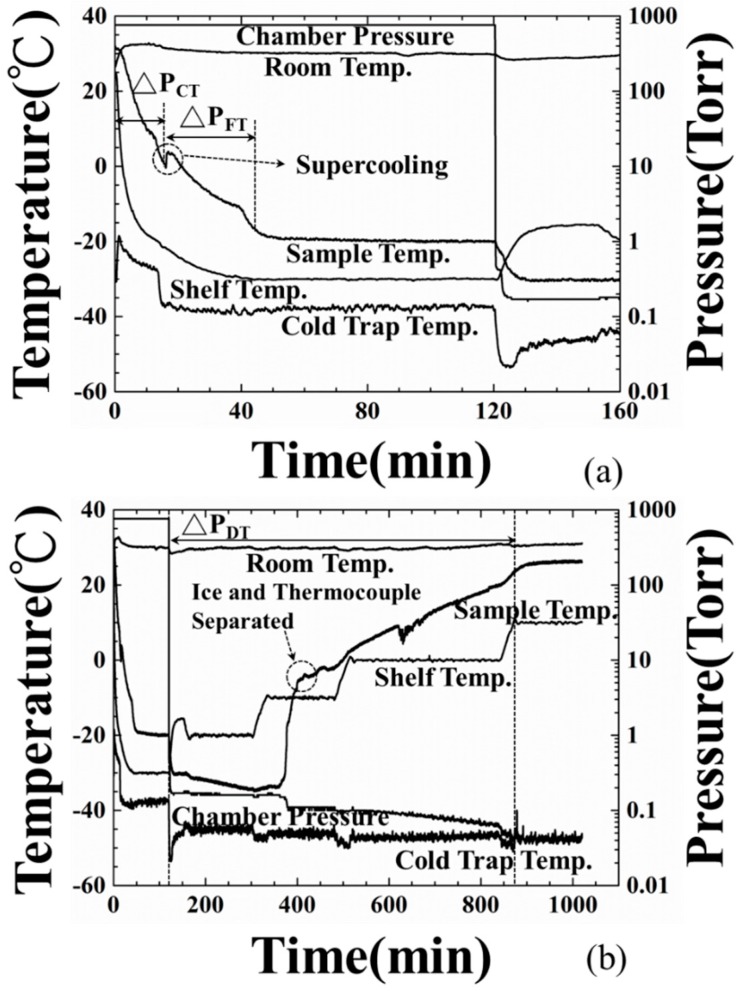
Temperature (room, shelf, sample, cold trap) and pressure (chamber) variations during freeze-drying process at (**a**) the freezing stage and (**b**) drying stage.

Temperature and pressure variations during the freeze-drying process have a close relationship with the state of the water/ice and both affect the size of the ice crystal nuclei and the freeze-dried product’s quality. In the experiment, the measured room, shelf, cold trap, water/ice temperatures and pressures for the freezing and drying periods of the freeze-drying procedure of the water at a level of 15 mm are shown in Figure 5a,b, respectively. In Figure 5a, the room temperature was 32 °C during the entire process and the chamber pressure was kept at 760 Torr for the experiment’s duration of 120 min. The shelf and cold trap temperatures were set at −30 and −40 °C and reached the desired values at 40 and 15 min, respectively. The water temperature was 32.1 °C in the beginning and was cooled down to a supercooled state of −0.4 °C, 16.1 min into the experiment. After the latent heat of the water was released completely, the phase change from water to ice was completed at 43.7 min and the ice temperature was −16.5 °C. Therefore, the cooling (ΔP_CT_) and freezing (ΔP_FT_) period determined by the sample temperature could be defined as the period from the start of cooling to the supercooled state and from the supercooled state to the phase change end, respectively. 

At the 120-min mark of the experiment, the drying procedure started and the chamber pressure was reduced from 760 to 0.17 Torr. The shelf temperature was increased in 10 °C increments, from −30 to 10 °C, according to the designed schedule in Table 1. The cold trap temperature was cooled down further to condense the vapor exhausted from the chamber. The chamber pressure was reduced further to 0.036 Torr during the drying process. At the 370-min mark, the ice temperature increased suddenly from −36 to −10 °C due to the sublimation of the ice and gradually increased to 23 °C by the end of the experiment. This abnormal situation was due to the detachment of the thermocouple from the ice and will be confirmed in the following section. Consequently, the ice temperatures during the second drying period is not available. The drying end could not be estimated from the temperature information and the typical experiment procedure would end at the 1020-min mark of the experiment. 

### 4.2. Amplitude Variation of Ultrasonic Signals during the Freeze-Drying Process

**Figure 6:**
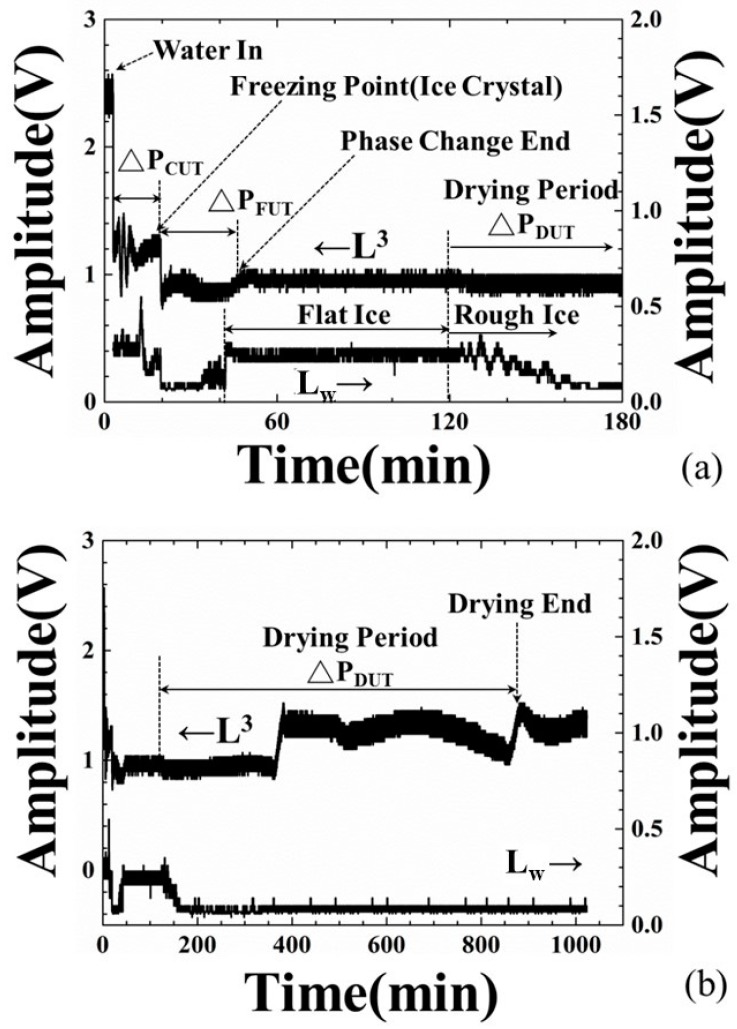
Amplitude variations of ultrasonic L^3^ and L_w_ echoes during freeze-drying process at (**a**) freezing stage and (**b**) drying stage, indicating the water-in, freezing point (ice crystal), phase change end and drying end.

The application of freeze-drying technology can guarantee the product’s quality. However, the process diagnosis and control will benefit by guaranteeing product quality and energy conservation. The conventional tools for monitoring this process may be limited to visual observation and temperature measurement. According to the authors’ knowledge, utilizing ultrasonic technology to monitor the freeze-drying process is rare. In order to investigate the correlation between the observed ultrasonic signals and the freeze-drying process of water, the amplitudes of the L^3^ and L_w_ echoes in Figure 3b with respect to the process time were acquired and presented in Figure 6. The details of freeze-drying process measured by the amplitude variations of ultrasonic L^3^ and L_w_ echoes were demonstrated as follows:

(1) At the 3.0-min mark: Water was poured into the container. At this moment, the amplitude of ultrasonic L^3^ echo decreased and L_w_ echo appeared, due to the fact that a part of the ultrasonic energy transmitted into the water through the steel plate/water interface. Water started to cool down.
(2) At the 19.2-min mark: The amplitudes of the L^3^ and L_w_ echoes decreased further, due to the variation of water acoustic impedance. In this supercooling period, this ultrasonic phenomenon may indicate the appearance of ice crystals. The alteration of water property will cause electrical impedance changing [32]. Hence, the amplitude decreasing point of the L^3^ and L_w_ echoes is defined as the freezing point of water and the cooling period (ΔP_CUT_) determined by the amplitude of the L^3^ echo is defined as the period from water-in to the freezing point.
(3) At the 43.0-min mark: The amplitude of the L^3^ echo increased to the relative maximum value and that of the L_w_ echo increased to a stable level, due to the stable ultrasonic impedance. This ultrasonic phenomenon may indicate phase change end of water and a flat ice surface. Hence, the freezing period (ΔP_FUT_) determined by the ultrasonic L^3^ echo is defined as the period from the freezing point to the phase change end.
(4) At the 125.0-min mark: The chamber was exhausted and the ice started to sublimate at the 120-min mark. The amplitude of L^3^ echo decreased a little and that of L_w_ echo disappeared gradually, due to the sublimation of ice and the rough ice surface.
(5) At the 370.0-min mark: The amplitude of the L^3^ echo increased suddenly from the bottom line and reach a stable value, due to the fact that the electromechanical coupling factor varied and the ultrasonic energy transmitting into the ice through the steel/ice interface reduced [33]. This ultrasonic phenomenon indicated the reduction of contact surface between ice and steel plate in the drying stage. At this moment, the sublimation of the ice reached a certain level and the thermocouple detached from the ice, as shown in Figure 5b.
(6) At the 885.6-min mark: The amplitude of the L^3^ echo increased suddenly from the reducing tendency and reached a stable value at this moment, due to the fact that the electromechanical coupling factor varied and ultrasonic energy transmitting into the ice through the steel/ice interface reduced further. This ultrasonic phenomenon indicated the complete sublimation of the ice and only few minerals remained on the steel plate surface. This moment is defined as the drying end point. The drying period (ΔP_DUT_) determined by the ultrasonic L^3^ echo is defined as the period from the exhaust of chamber to the complete sublimation of the ice, that is, the drying end point. Compared with the typical experiment settings, the indication of drying ends for water by the amplitude variations of ultrasonic L^3^ echo could reduce the processing period of the 134.4 min/cycle and save 13% of consumed electricity.

### 4.3. Variation of Ultrasonic Velocity during the Freeze-Drying Process

**Figure 7:**
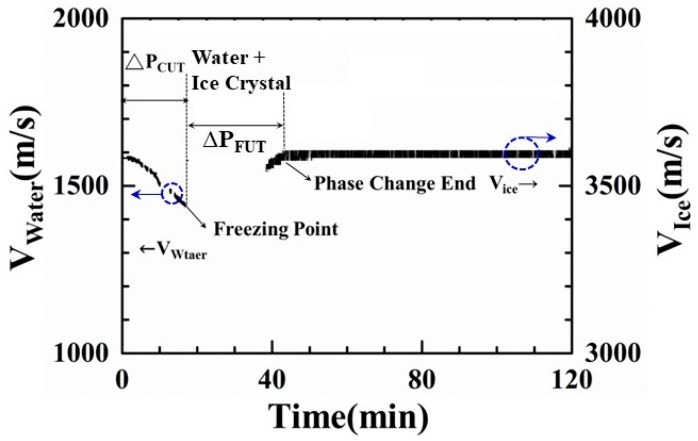
Ultrasonic velocity variation in the water during the freeze-drying process, which indicates the water-in, the freezing point (ice crystallization) and the phase change end.

During the freezing process, the slow cooling speed caused bigger ice crystal nuclei than did the fast cooling time. Ultrasonic velocity has a close relationship with temperature and it is one of the indicators that shows the cooling speed of water, the phase change end and the start of sublimation. The ultrasonic velocity in the water is calculated according to the following equation:

(1) $$v_{w}\;=\;2H/\Delta t$$

where H is the height of the water level in Figure 3a and Δt is the time delay between the ultrasonic L^1^ and L_w_ echoes in Figure 3b. The result is shown in Figure 7.

In Figure 7, when the water was poured into the container, the ultrasonic velocity was first measured at the 3.0-min mark under the water temperature of 27.2 °C. At that moment, the ultrasonic velocity in the water was 1588.5 m/s. From the 3.0 to the 17.4-min mark, when the water temperature decreased to 1.0 °C, the ultrasonic velocity decreased to 1434.5 m/s, indicating that the temperature of the water had decreased during the cooling process. Comparing Figure 7 with Figure 5a, the relationship between ultrasonic velocity and water temperature is expressed by the following equation.

(2) $$v_{w}\;\left(T_{w}\right)\;=\;1.42418\times 10^{3}+1.03190\times 10\times T_{w}-0.15453\times T_{w}^{2}$$

where v_w_ (m/s) is the ultrasonic velocity in water and T_w_ is the water temperature. The ultrasonic velocity decreased with the reducing water temperature. The ultrasonic velocity presented by authors in this study is higher than that presented by Bilanuik and Wang [34], due to some minerals contained in the water. Therefore, the cooling speed of water can be determined by measuring the ultrasonic velocity in the water. From the 17.4 to 38.3-min mark of the experiment, the ultrasonic velocity disappeared and the amplitude of the L_w_ echo also decreased in noise level due to the formation of ice crystal nuclei. After the 38.3-min mark, the ultrasonic L_w_ echo appeared again and the ultrasonic velocity gradually increased from 3586.4 to 3597.2 m/s, due to the phase change end of the water and the completion of the freezing process. From 43.0 to 120.0-min mark, the ultrasonic velocity maintained a steady level of 3597.2 m/s for the pausing period. Therefore, ultrasonic velocity can be used to indicate the cooling speed, the phase change end and completion of the freezing process of water.

### 4.4. Visual Observation of the Container during the Freeze-Drying Process

**Figure 8:**
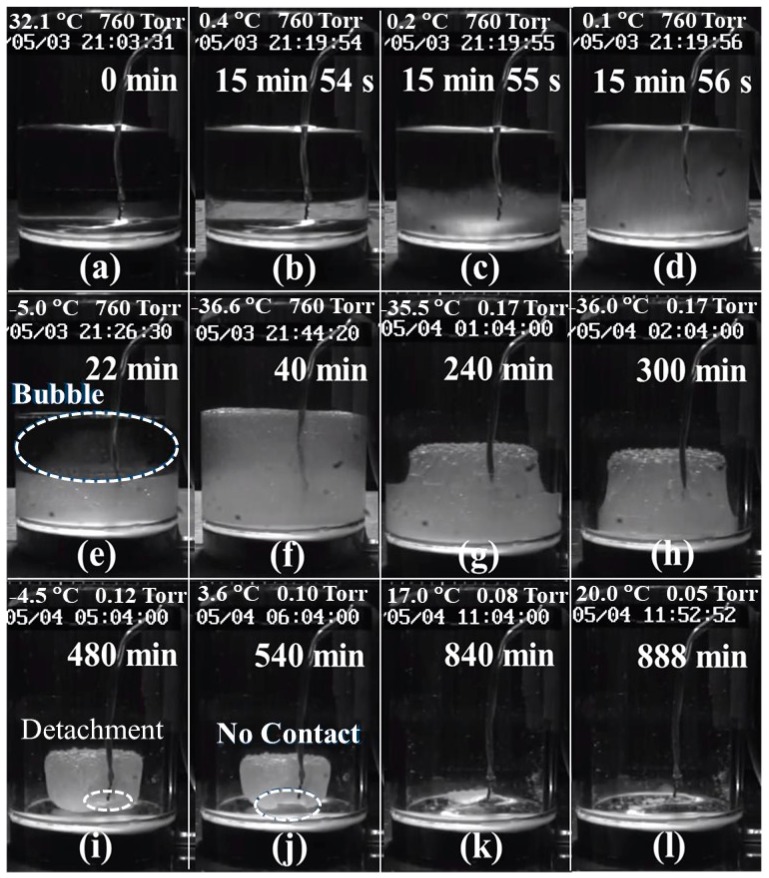
Photographs of the water/ice in the container during the freeze-drying process along with the corresponding process times of (**a**) 0 min, (**b**–**d**) 15 min 54~56 s, (**e**) 22 min, (**f**) 40 min, (**g**) 240 min, (**h**) 300 min, (**i**) 480 min, (**j**) 540 min, (**k**) 840 min and (**l**) 888 min.

The dynamic physical phenomena that can be observed in the container shows the phase change from water to ice and the sublimation detail of the ice during the freeze-drying process in great detail. These phenomena could also be evidence of other measured parameters. Figure 8a–l are the photographs of the container with water/ice during the freeze-drying process at the 0–888-min mark, respectively. The freeze-drying process observed in Figure 8a–l is described as follows:

(1) Figure 8a: Water was poured into the container that had a thermocouple in the middle and then the container was set on the shelf of the freeze-drying machine.
(2) Figure 8b–d: Most of the water was still in a liquid state. However, the water close to the bottom of the container started to become opaque and some ice crystals began to form beside the thermocouple. The ice crystals increased gradually from the bottom to the top of the container. This was an indication that the water started to freeze.
(3) Figure 8e: Half of the water became very slushy and the visibility became worse. Some air bubbles also appeared within the slush.
(4) Figure 8f: Most of the water had become frozen into ice. Some air bubbles were pushed up from the bottom to the top. The ice surface was flat, which indicated that the stress on the ice during the freezing process was reduced.
(5) Figure 8g–h: The chamber was exhausted and a vacuum was created. The ice sublimated from the top progressing downwards towards the bottom of the container and from the exterior inwards towards the interior of the container. The ice surface was composed of porous structure and appeared rough.
(6) Figure 8i–j: The ice shrank more due to further sublimation. The contact surface between the ice and the steel plate was also reduced. The thermocouple seemed to detach from the ice at the 480-min mark of the process. This could explain why the sample temperature increased suddenly from −36 to −10 °C at 390.5-min mark. At the 540-min mark, the contact surface between the ice and the steel plate was reduced even further, which may diminish the conduction of heat being transferred from the shelf to the ice.
(7) Figure 8k–l: There was tiny amount of residual ice that remained on the surface of the steel plate at the 840-min mark. Finally, at the 888-min mark, only a little of ice crystals remained on the surface of the steel plate.

The freeze-drying process can be observed clearly in Figure 8. These results can provide additional evidence for the previous measuring methods. However, this observing method is only suitable for the transparent container and plain solution. For the unobservable container and the opaque material, such as metal container and colored samples, the temperature and ultrasonic techniques are more capable to diagnose the mentioned phenomena. From the measured results, the thermocouple detached from the ice and further temperature information of the ice was not available. In this viewpoint, the ultrasonic technique is a more reliable and capable method for diagnosing the freeze-drying process.

### 4.5. Freeze-Drying Processes of Various Water Levels

**Figure 9:**
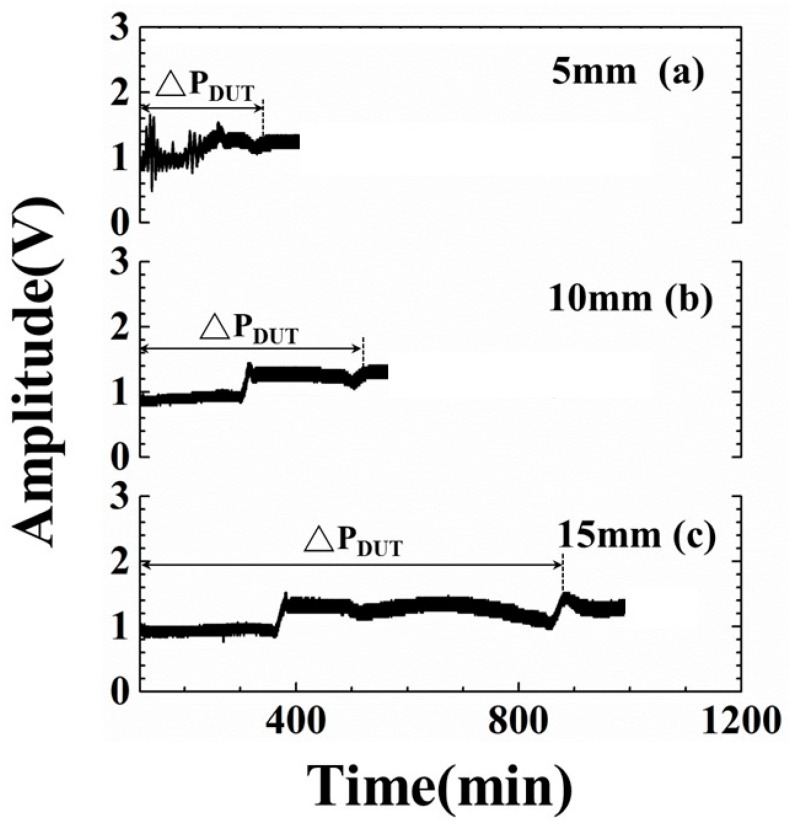
Amplitude variations of ultrasonic L^3^ echo with respect to various water levels at drying stage. Water level: (**a**) 5, (**b**) 10 and (**c**) 15 mm.

Before applying the ultrasonic technology to the freeze-drying process, the linear relationship between ultrasonic signatures and the water level is the fundamental requirement. To verify this relationship, the containers, which were filled with water in the height of 5, 10, 15 mm, passed through the freeze-drying process under the shelf temperature setting of −30 °C. In this research, the variation during the drying period will be evaluated. During the drying stage, the thermocouple cannot measure the samples’ temperature profiles due to the detachment of thermocouple from the sample. The ultrasonic signals are able to diagnose the drying phenomena. The experimental results of the amplitude of the ultrasonic L^3^ echo are shown in Figure 9. Figure 9a–c are the amplitude variations of ultrasonic L^3^ echo in water levels of 5, 10, 15 mm, respectively, during the drying stage. The timings of the drying end indicated by ultrasonic L^3^ echo are 329.8, 526.7 and 885.6-min mark for the water levels of 5, 10 and 15 mm, respectively. The drying periods of various water levels are also indicated. The corresponding timings of the drying end indicated by ultrasonic L^3^ echo are illustrated in Table 2. It seems that there is a linear relationship between the timings of drying end and the water level.

**Figure 10:**
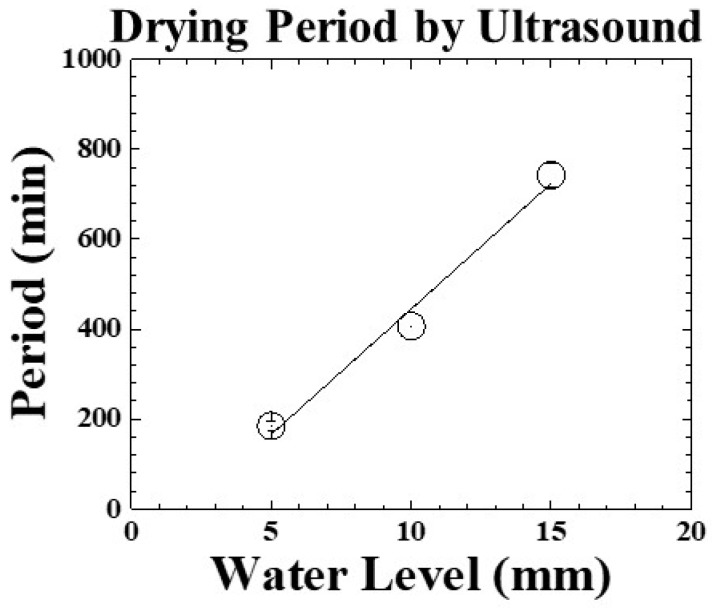
Drying periods measured by ultrasonic signal with respect to various water levels. Water level: 5, 10, 15 mm.

The measured drying periods are compared with the water level for evaluating the mentioned linear relationship. The results are shown in Figure 10. The estimated error of drying period under the experimental conditions is less than 5%. In the water level range from 5 to 15 mm, the average drying periods indicated by ultrasonic L^3^ echo increase from 185.1 to 742.8 min. The slope of the fitting line is 55.8 min/mm. The drying period is expressed as:

(3) $$\Delta P_{DUT}=-112.8+55.8*H$$

where ΔP_DUT_ is the drying period in Figure 9 and H is the water level in Figure 3. Even though there are no temperature and visual information, one can still estimate the required drying period according to the filled water level based on Equation (3). In the future, this ultrasonic sensor and evaluating technology would be installed into freeze drying machine to detect the phase change and feedback the information to machine control system, as shown in Figure 1. Comparing with the typical experiment settings of 1020 min, the ultrasonic technique can clearly indicate the dynamic phenomena and completion of cooling/freezing/drying stages at each water level before the end of the experiment for reducing the freeze-drying process and saving energy.

## 5. Conclusions

In this paper, an ultrasonic transducer (UT) is integrated onto the bottom of a specially designed container to analyze the freeze-drying process of water at various water amounts. The measured ultrasonic signatures are compared with the temperature and visual records. Among these three measured methods, ultrasonic and visual records are able to document the entire progression of the freeze-drying process, including the water-in, freezing/drying points, the phase change end of the water and the cooling/freezing/drying periods. The ultrasonic velocity in the water also indicates the cooling tendency of the water. The drying period increases with the water level. The increase rate, which is evaluated by the amplitude of the ultrasonic L^3^ echo, is 55.8 min/mm. During the drying stage, the thermocouple cannot measure the entire temperature profile of the ice due to the detachment of the thermocouple from the ice. Only the ultrasonic signals and visual records are utilized for diagnosing the drying phenomena. However, the observing method is only suitable for the transparent container and plain solution. Comparing with the typical experiment settings, the indication of drying ends for water at level of 15 mm by the amplitude variations of ultrasonic L^3^ echo could reduce the process period of 134.4 min/cycle and save 13% of consumed electricity. Therefore, this study demonstrates the use of a specially designed container, integrated ultrasonic sensor and technology for analyzing and optimizing the freeze-drying process of water for saving the process cost and energy.

## Figures and Tables

**Table 1:** The typical procedure used for the freeze-drying process.

Freezing Temp. (°C)	Freezing Period (min)	Chamber Pressure (Torr)	Drying Temp. (°C)	Heating Period (min)	Pausing Period (min)
−30	120	760 | 0.17	−20	60	120
			−10	60	120
			0	60	300
			10	60	120

**Table 2:** Timings and temperatures of water in, freezing point, phase change end and drying end indicated by temperature and ultrasonic L^3^ echo for water levels of 5, 10, 15 mm.

Water Level (mm)	Items	Watering Point	Freezing Point	Phase Change End	Drying End
**5**	Time_Temp_ (min)	3.0	16.3	24.4	N/A
	Time_UT_ (min)	3.0	15.8	20.6	329.8
	Temperature (°C)	29.8	−2.8	−10.8	N/A
**10**	Time_Temp_ (min)	3.0	14.0	28.9	N/A
	Time_UT_ (min)	3.0	13.7	27.1	526.7
	Temperature (°C)	30.4	−2.4	−14.9	N/A
**15**	Time_Temp_ (min)	3.0	19.2	46.7	N/A
	Time_UT_ (min)	3.0	19.5	45.8	885.6
	Temperature (°C)	32.1	−0.4	−16.5	N/A
